# A Light-Activated Explosive Micropropeller

**DOI:** 10.1038/s41598-017-04908-x

**Published:** 2017-07-04

**Authors:** Qianlan Rao, Tieyan Si, Zhiguang Wu, Mingjun Xuan, Qiang He

**Affiliations:** 1 Key Laboratory of Microsystems and Microstructures Manufacturing, Micro/Nanotechnology Research Center, Harbin Institute of Technology, Yikuangjie 2, Harbin, 150080 China; 2 Max Planck Institute for Intelligent Systems, Heisenbergstrasse 3, 70569 Stuttgart, Germany

## Abstract

Self-propelled micro/nanomotors possess tremendous exciting promise in diverse fields. We describe an asymmetric, fuel-free and near-infrared light-powered torpedo micromotor, which is constructed by using a porous membrane-assisted layer-by-layer sol-gel method to form silica multilayer inside the pores, following by the deposition of gold nanoparticles on one end of the pores. In the absence of chemical fuels, the high propulsion of microtorpedoes under illumination of near-infrared light is owing to the photo-thermal effect of gold clusters, generating a thermal gradient inside the microtorpedoes. The speed of microtorpedoes is dependent on the laser powers and media. More interestingly, such fuel free-powered microtorpedoes could explode triggered by higher laser power at the predefined site and thus provide a new platform for future biomedical applications.

## Introduction

The development of artificial micro/nanomotors launched a new horizon in the field of nanotechnology as their potential promises for environmental remediation, dynamic assembly of intelligent materials, precise disease treatment, and lab-on-chip devices^1–14^. By mimicking autonomous movement of chemically-powered natural biomolecular motors, various artificial micro/nanomotors have been brought to the foreground over past decade, leading to the development of chemical fuels or other physically actuated motors^15–21^. Various chemicals, such as hydrogen peroxide^22–26^, hydrazine^27^, acid/based^28, 29^, and bromine/iodide^30^ provide chemical energy for the self-propulsion of synthetic motors. Despite considerable progress in chemically-powered micro/nanomotors has been achieved, one must be taken into attention that most of chemical fuels are not segmental environment friendly and their relative toxicity is biologically incompatible. To avoid the toxicity of chemical fuels, physical stimulus-powered micro/nanomotors are under intense research recently. Ultrasonic, magnetic field, electric field, and light illumination had been successfully developed to propel micro/nanomotors in the absence of chemical fuels^31–36^. Among various physical triggers to actuate the propulsion of synthetic motors, near infra-red light (NIR) attracts considerable attention owing to its optimal penetration and minimum absorption in biological tissues, showing promising potential applications in the field of biomedicines^37^. Previously we reported the NIR laser-triggered propulsion of the polymer multilayer microrocket through thermophesis^10^. Due to the limited illuminating area and ununiform density of NIR laser, the movement behavior of such polymer rocket is not stable and not easy to achieve the for the long term.

Here we demonstrate the successful construction of gold-functionalized torpedo micromotors based on a porous membrane-assisted layer-by-layer sol-gel method, following the assembly of gold clusters in the big opening (tail) of the microtorpedoes. Under the irradiation of NIR light, the microtorpedoes swim along a rather straight trajectory under the propulsion of self-thermophoretic force as physically simulated^38, 39^. Compared with the previous polymer multilayer rocket, the torpedo micromotors possess gold nanoparticles layer which are mainly located around the large opening of torpedo micromotors. Addtionally, the two-photon confocal laser scanning microscope provide an advanced light source which sustains with an equally photon-distributed and average-powered focus plane. The combination of torpedo design and improved light setup accomplished the NIR light-triggered high movement with stable behaviour. The movement of the microtorpedo can be modulated by varying the power of NIR light, and also the torpedoes displays efficient movement in various fluids such as pure water, salt solution, PBS, and cell cultured medium. More interestingly, these microtorpedoes could instantaneously explode to several pieces upon a high NIR power. The computational simulation reveals that the NIR irradiation onto the torpedo rapidly increase the temperature, leading to intensive evaporation inside the torpedo micromotor and the following explosion of the torpedo. Such a light-driven microtorpedo with an explosive capability offers a new possibility for the application of fuel-free micro/nanomachines in biomedical field.

## Results and Discussion

**Figure 1:**
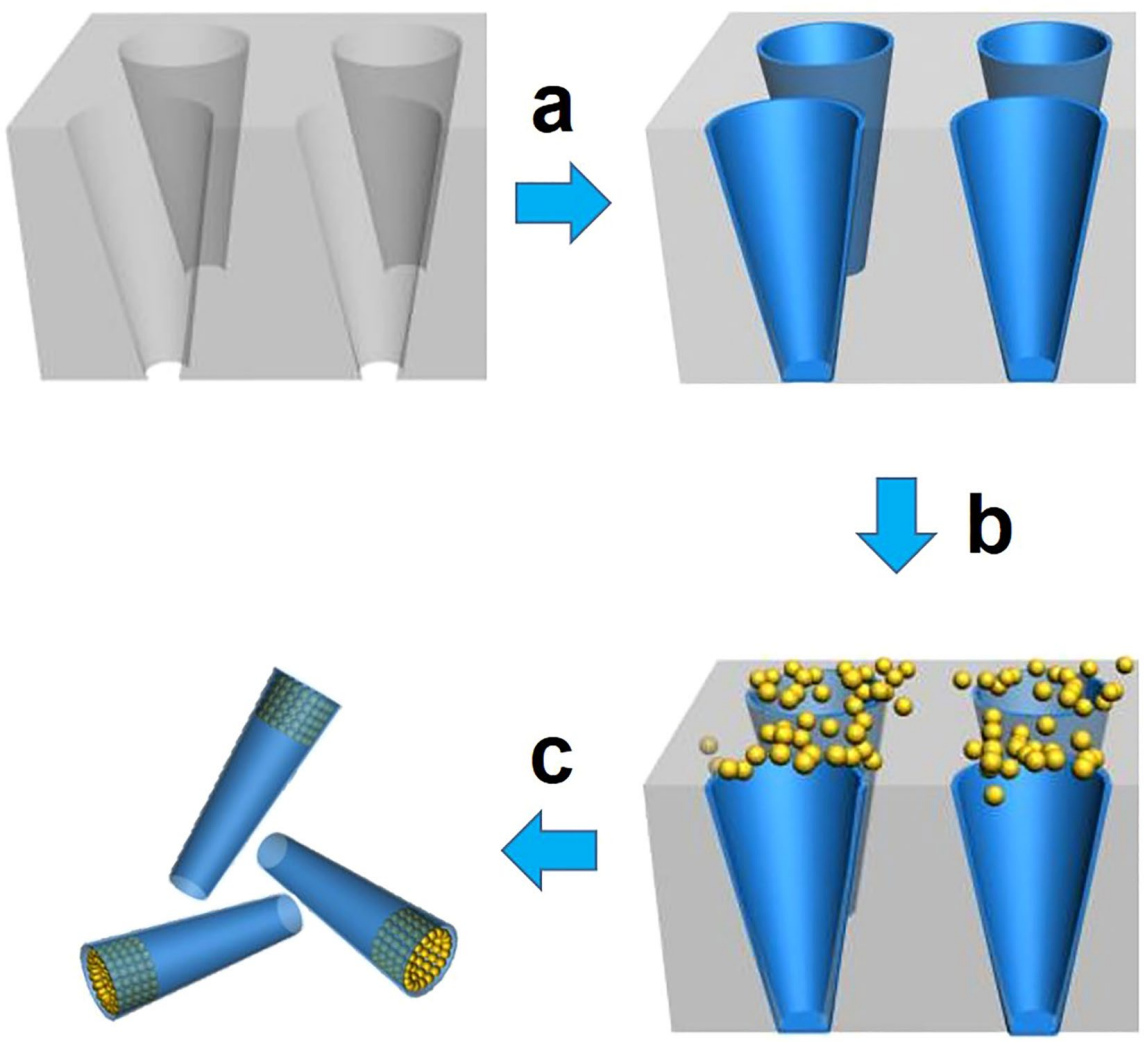
Fabrication Process of the Gold Film-functionalized Torpedo Motor. (**a**) Deposition of 3 layers of silica via the sol-gel method. (**b**) Deposition of Au nanoparticles to the big opening of torpedo motor. (**c**) Dissolution of the porous template to obtain the resulting torpedo.

The fabrication strategy is schematically illustrated in Fig. 1. Three layers of silica as a framework were firstly deposited by applying a porous membrane-assisted layer-by-layer sol-gel method (SSG). Briefly, the SiCl_4_ as a silica precursor was deposited into the pores of a polycarbonate membrane (pore size: 3 μm) with a thickness of 8 ± 2 μm, and then the SiCl_4_ was hydrated to one layer of silica inside the pores by immersing the membrane into water. After 3 layers of silica were deposited, the citrate-stabilized gold nanoparticles (AuNPs) were integrated into the pores. It should be noted that the AuNPs were aggregated into gold clusters around the big opening of silica torpedoes by using a repeated wet-dry process^40, 41^. To improve the connetion of the silica layers between the pores and the surface of the template, a polishing step onto the surface of the template was conducted with wet cotton containing alumina powder (average mech size of 30 μm). Finally, the torpedo motors with gold clusters were collected by dissolving the membrane with CH_2_Cl_2_.

**Figure 2:**
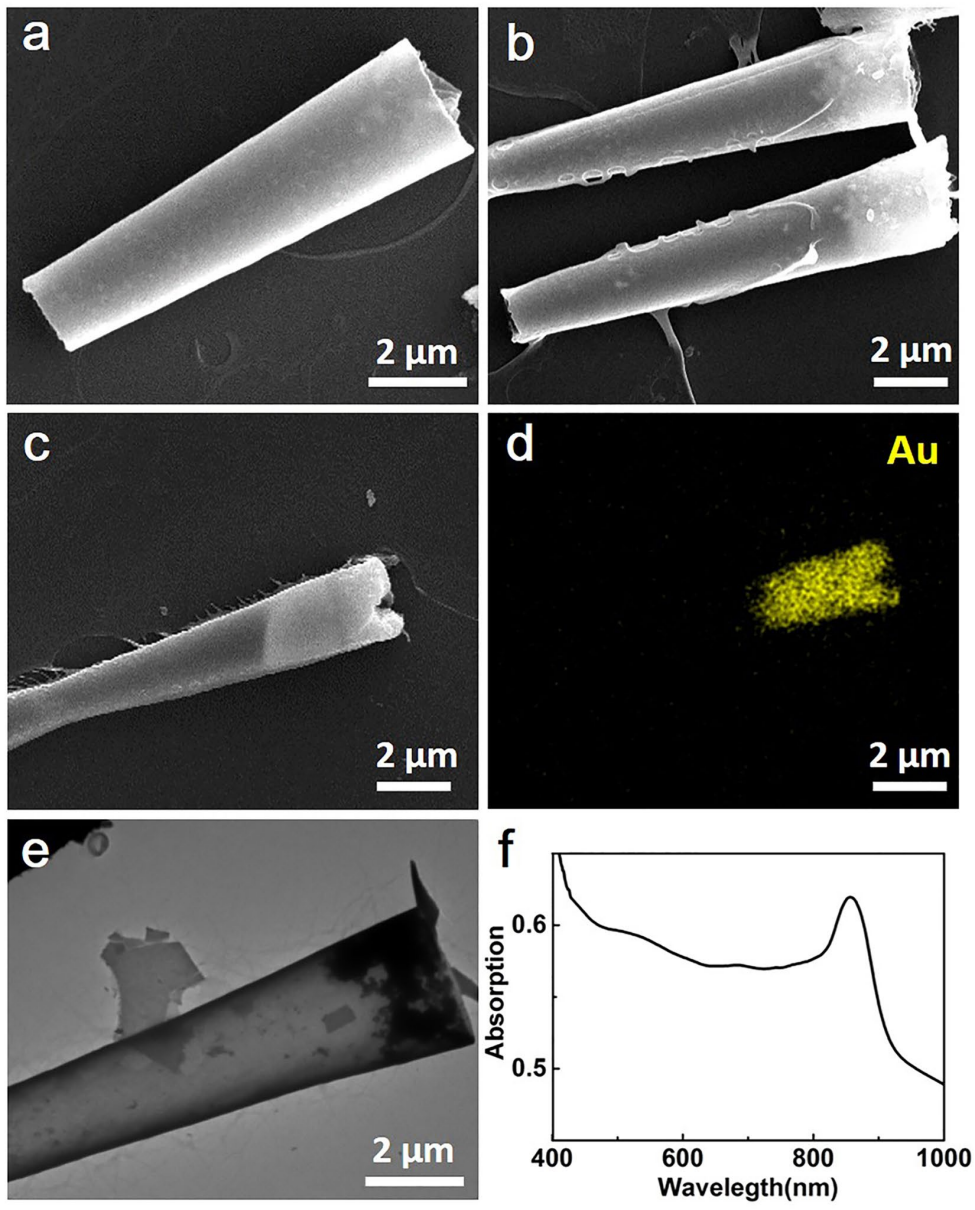
Characterization of the Gold Film-functionalized Torpedoes Motor. (**a**) SEM image of bare torpedoes without Au nanoparticles. (**b**) SEM image of Au-tailed torpedo. (**c**,**d**) SEM image of Au-tailed torpedo and its corresponding Au EDX mapping. (**e**) TEM image of Au-tailed torpedo. (**f**) UV/Vis-NIR extinction spectra of Au-tailed microtorpedoes.

The scanning electron microscopy (SEM) image in Fig. 2a shows the asymmetric tubular geometry of a bare silica torpedo without AuNPs. The silica framework is smoother than our previous polymer multilayer tubes^22^. The length is roughly 8 ± 2 μm in accordance of thickness of the template, and the size of wide opening and narrow opening are 3 ± 0.5 μm and 1.5 ± 0.5 μm. The top view SEM image in Supplementary Fig. S1 illustrates that the thickness of the bare torpedo is about 105 ± 15 nm, representing a 35 ± 5 nm per layer. This bare torpedo is thicker than the previously reported silica tubes with same number of layers^42^. It should be attributed to the prolonged adsorption and hydrolysis time of SiCl_4_. Compared with the bare silica torpedoes, the AuNPs-functionalized torpedoes reserve the geometry and size of bare torpedo well, and the bright region is ascribed to the integration of gold clusters (Fig. 2b). An energy dispersive X-ray (EDX) mapping analysis was carried out to investigate the gold distribution of the microtorpedoes (Fig. 2c,d). Thebright yellow region in Fig. 2d displays the distribution of AuNPs clusters mainly located in the wide opening of the torpedo. The transmission electron microscopy (TEM) image in Fig. 2e further confirms the distribution of AuNPs clusters in the big opening. More interestingly, the UV-vis-NIR spectrum in Fig. 2f indicates the maximum adsorption peak of the AuNPs-functionalized torpedoes is approximately 850 nm, which is essential for the NIR light-triggered propulsion.

**Figure 3:**
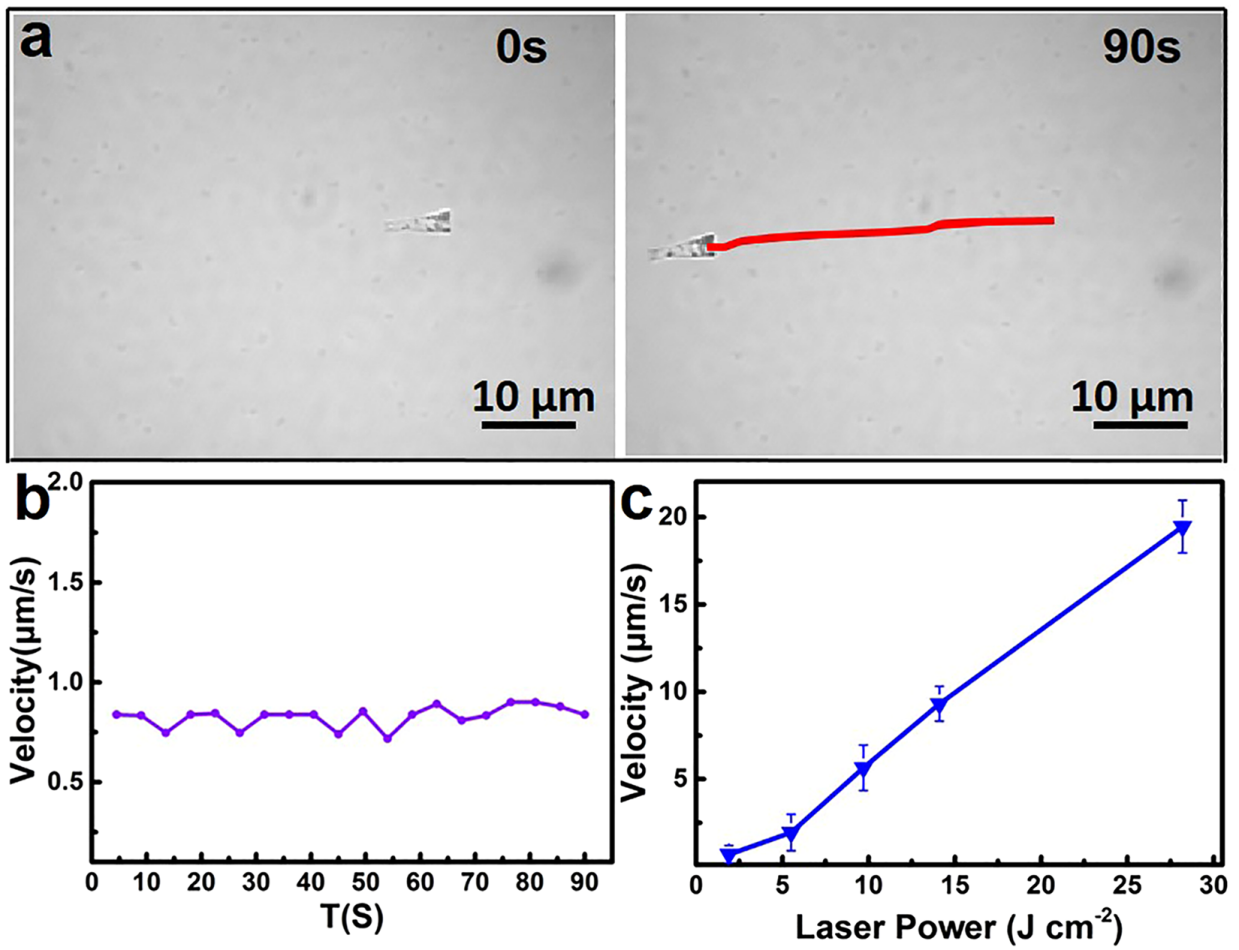
The Movement Behavior of  NIR-Propelled Torpedo Motors. (**a**) Time-lapsed images of the torpedo under the NIR irradiation with wavelength of 808 nm in pure water (laser power: 5.5 J cm^−2^). The red line represents the tracking trajectory of the torpedoes. (**b**) The corresponding velocity of the torpedoes in (**a**) as the function of time. (**c**) The maximal velocity in 1.5 s of ASRs with laser power of 1.9 J cm^−2^, 5.5 J cm^−2^, 9.7 J cm^−2^, 14.1 J cm^−2^, 28.2 J cm^−2^.

In order to illuminate the torpedoes, NIR laser beam was vertically guided onto the sample plane through an objective lens of a two-photon confocal laser scanning microscope. The time-lapse images in Fig. 3a captured from Supplementary Video 1, illustrate a nearly linear trajectory of the torpedoes under the NIR irradiation with a power of 5.5 J cm^−2^ as indicated by the red line. The direction of motion is along the body axis from the wide opening to the narrow opening. Similar with previous reports^32^, the motion of microtorpedoes should also be ascribed that the torpedo motors convert the adsorbed photons to heat owing to the plasma resonance adsorption of AuNPs clusters in the NIR region. The AuNPs clusters covering the wide opening and asymmetric tubular geometry of the torpedoes under NIR irradiation result in an asymmetric thermal gradient, generating athermophoreticforce along the body axis. Figure 3b records the speed distribution of the torpedo in Fig. 3a. The microtorpedo motor displays a straight movement with a stable speed of 0.8 μm/s during 90 s. Unlike our previous researches, a fixed, focused laser dot made the velocity of torpedo motors increasing firstly and subsequently decreasing^22, 23^. We here used the two-photon confocal laser scanning microscope (TP-CLSM) combined with the temporal concentration femtosecond ultrafast pulses laser. It is an equally photon-distributed and average-powered focus plane, like a defocus laser irradiation. The real-time monitoring technology in TP-CLSM also enhances system reliability by providing excellent beam stability and minimal power fluctuations during the irradiation duration. The velocity of the torpedo could be easily controlled by modulating NIR laser power as shown in Fig. 3c. The maximum velocity at a laser power of 28.2 J cm^−2^ is 19.5 ± 1.5 μm s^−1^. A linear relationship between the NIR laser power and velocity offers a convenient and quantitative control upon the torpedo motor, including the on-demand deceleration, acceleration, and controlled velocity.

Theoretical simulation was conducted to investigate the photo-thermal effect and the propulsion mechanism of torpedo motors under NIR irradiation. The tail of the asymmetric torpedo is covered by cylindrical AuNPs clusters. The AuNPs clusters convert the absorbed photons to heat, acting as a heating source. Accordingly, the kinetic energy of water molecules around the AuNPs clusters-functionalized tail is improved greatly. This thermophoretic force is proportional to the local temperature gradient around the AuNPs clusters-functionalized tail^43–45^,

1 $${{\rm{F}}}_{{\rm{thermophoresis}}}=C{\rm{\Delta }}T$$

Here the coefficient:

2 $${\rm{C}}=\frac{9\pi {\rm{p}}{\eta }^{2}{\rm{Ka}}}{2\rho {{\rm{gTk}}}_{{\rm{p}}}}$$

**Figure 4:**
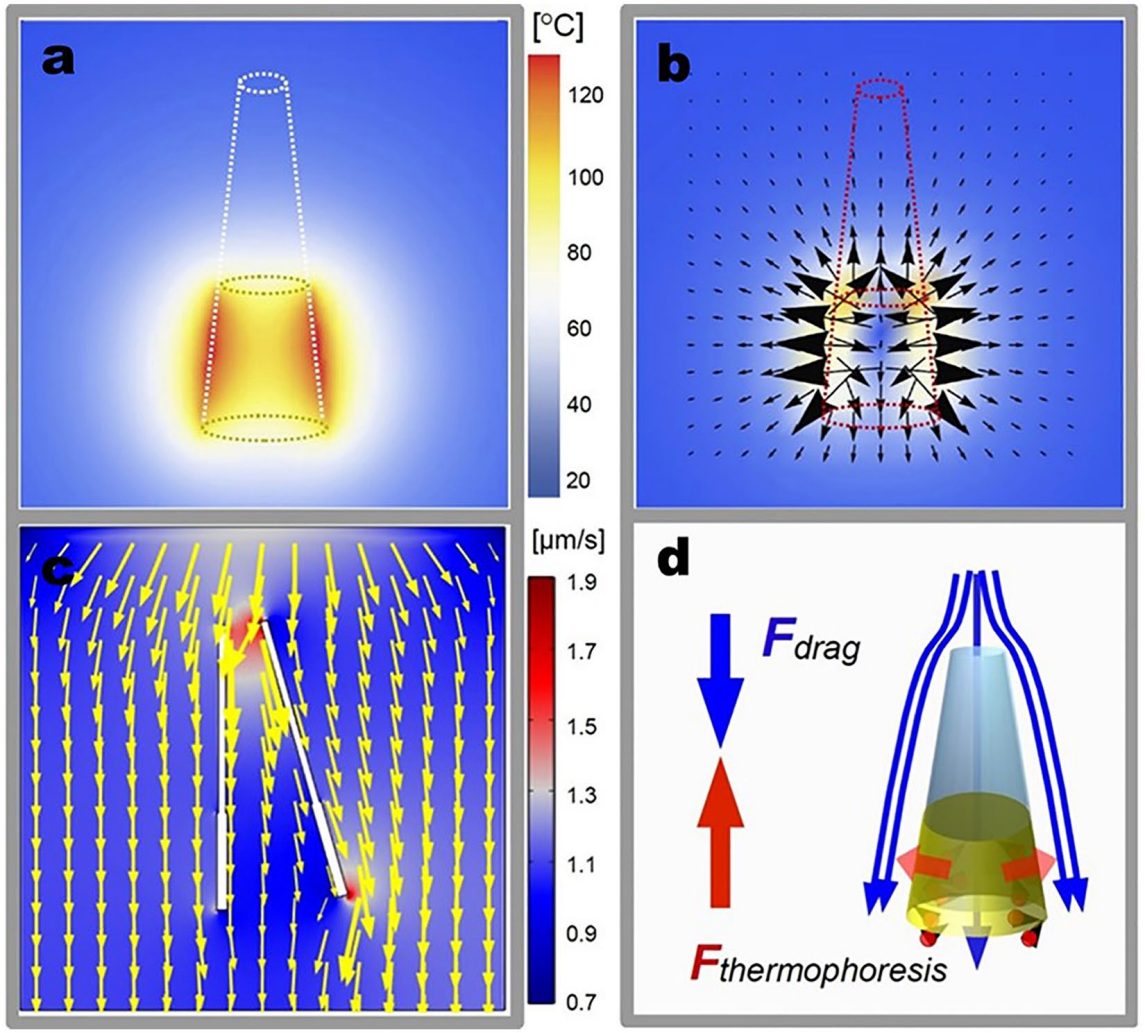
Theoretical Simulation of the NIR-Driven Propulsion of the Torpedo Motor. (**a**) The local temperature around the asymmetric torpedo with its tail covered by Au film on both inner wall and outside wall. (**b**) The computed thermophoresis force distribution around the cylinder Au shell, F_thermophoresis_. The black arrows indicates the force vectors, its size is proportional to the strength of the force. The degree of the color indicated absolute value of the force at that spatial point. (**c**) The velocity field distribution of fluid around a moving torpedo. The maximum velocity is around the narrow mouth and the tip of the wide tail. (**d**) The scheme of forces acting on the torpedo. The thermophoresis force (the red arrows pointing upward) pushes torpedo forward. While the drag force (the blue arrows pointing upward) due to viscous force of aqueous environment slows down the torpedo.

The computed temperature around AuNPs clusters by the heat diffusion equation is shown in Fig. 4a. The thermophoretic force is proportional to the square of the steam gas viscosity η, the radius of torpedo tail, and the ratio of the thermal conductivity of steam gas K_a_ to the thermal conductivity of gold k_p_. When the torpedo finally reaches a steady motion, the thermophoretic force is fully counterbalanced by the viscous resistant force. Figure 4b suggests that the strongest thermophoretic force vectors indicating by black arrows fall on the AuNPs clusters and point upward. Some weak force vectors generated by the hot steam gas layer inside the torpedo point to the opposite direction. The resultant vector of all of these independent force vectors point upward to push the torpedo forward. We further simplify the geometry of the torpedo motor as a cylinder-shell by neglecting the small difference of the radius between the small opening and big opening. The viscous resistant force due to viscous interaction in aqueous environment is computed by equation^46^,

3 $${{\rm{F}}}_{viscous}=\frac{2\pi \eta Lv}{\mathrm{ln}\,{(\frac{L}{R})}^{-0.72}}$$

Given that the aqueous media near the interface of gold film exist a hot steam in the scope of laser, and the speed of the torpedo maintains the stable speed of 0.8 μm/s. The steam dynamic viscosity η here is η = 1.29779*10^−5^ kg/ms. The length of torpedo (L) is 7 μm. The radius of the torpedo (R) is 2.25 μm. The computed viscous force with these parameters is 1.39 nN (F_viscous_ in Fig. 4d). Since the torpedo possesses a narrow opening on one end and a wide opening on the other end, this asymmetric geometry ensured a well-defined directional motion as demonstrated in Fig. 3a. To better understand the motion mechanism, the fluid dynamic simulation was used to Fig. out the velocity field distribution of fluid for a tilted torpedo (Fig. 4c). It can be seen that the flow passing through the central line of the torpedo enhances the robustness of directional motion. When the tail of the torpedo tilted to the right hand side, the flows on the right hand side point to the left hand side to exert a stronger impulse on the torpedo. This impulse force pushes the wide opening of the torpedo to the vertical axis.

**Figure 5:**
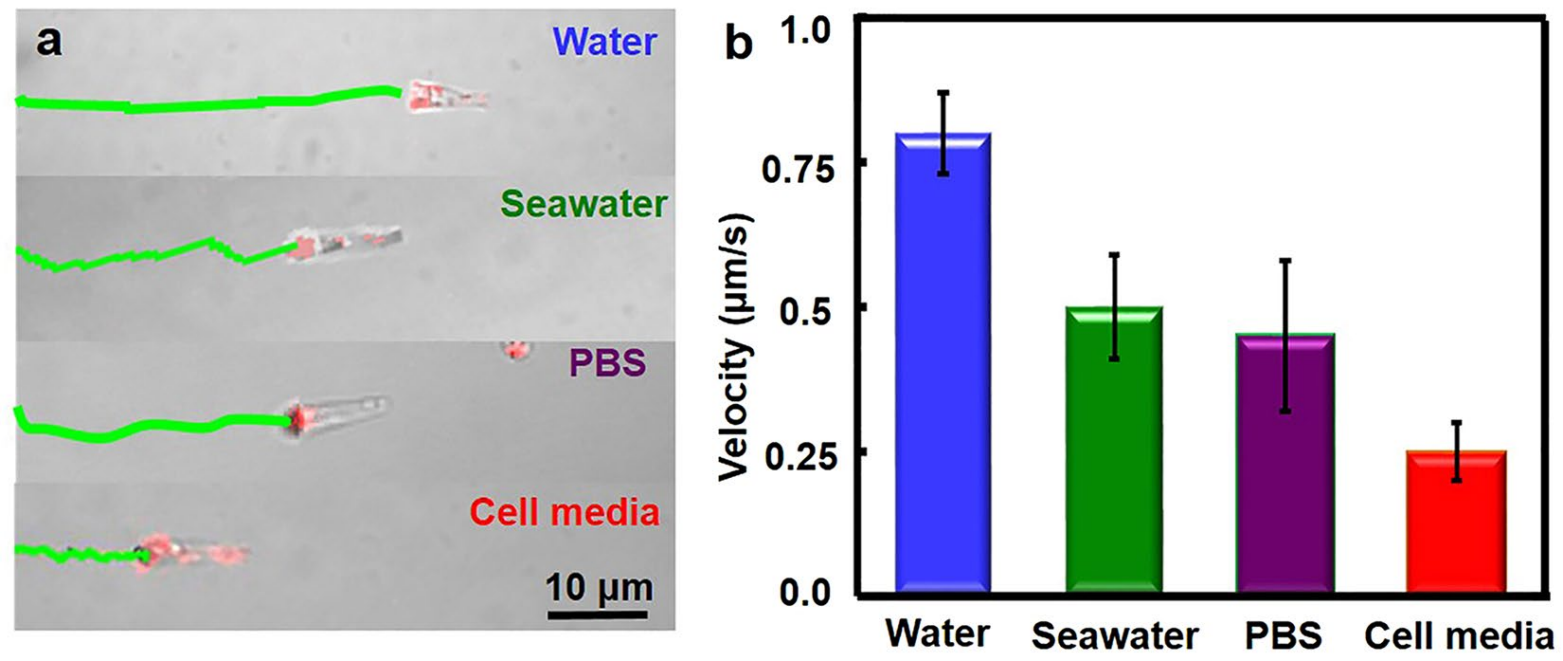
Torpedo Motors Move in Different Media. (**a**) The images showing the NIR movement of the torpedo motors in water, salt solution, PBS, and cell media. (**b**) Speed of the torpedo upon the 5.5 J cm^−2^ laser power in different media.

To accomplish complex tasks for future biomedical application, the motion of torpedoes was examined in diverse media. Figure 5a, captured from Supplementary Videos 2–4, shows the time-lapse images with track lines for the NIR-powered movement in 30 s, indicating that the NIR-activated torpedoes could efficiently move in pure water, PBS, seawater, and cell culture media. Also, the track lines with different distances under the same windows reflect the increased environmental viscosity of these fluids. The average speed during 30 s is roughly 0.8 μm/s, 0.5 μm/s, 0.4 μm/s and 0.3 μm/s, respectively (Fig. 5b). The decrease of velocity is owing to the increasing viscosity^33^. On the other hand, salts or proteins in close proximity to the AuNPs clusters could absorb thermal energy, resulting in lower velocity in complex media^47^. Note that the AuNPs-functionalized silica torpedo displays red fluorescence from AuNPs upon the irradiation of NIR laser owing to the surface plasmon resonance as shown in Fig. 5a ^48^.

**Figure 6:**
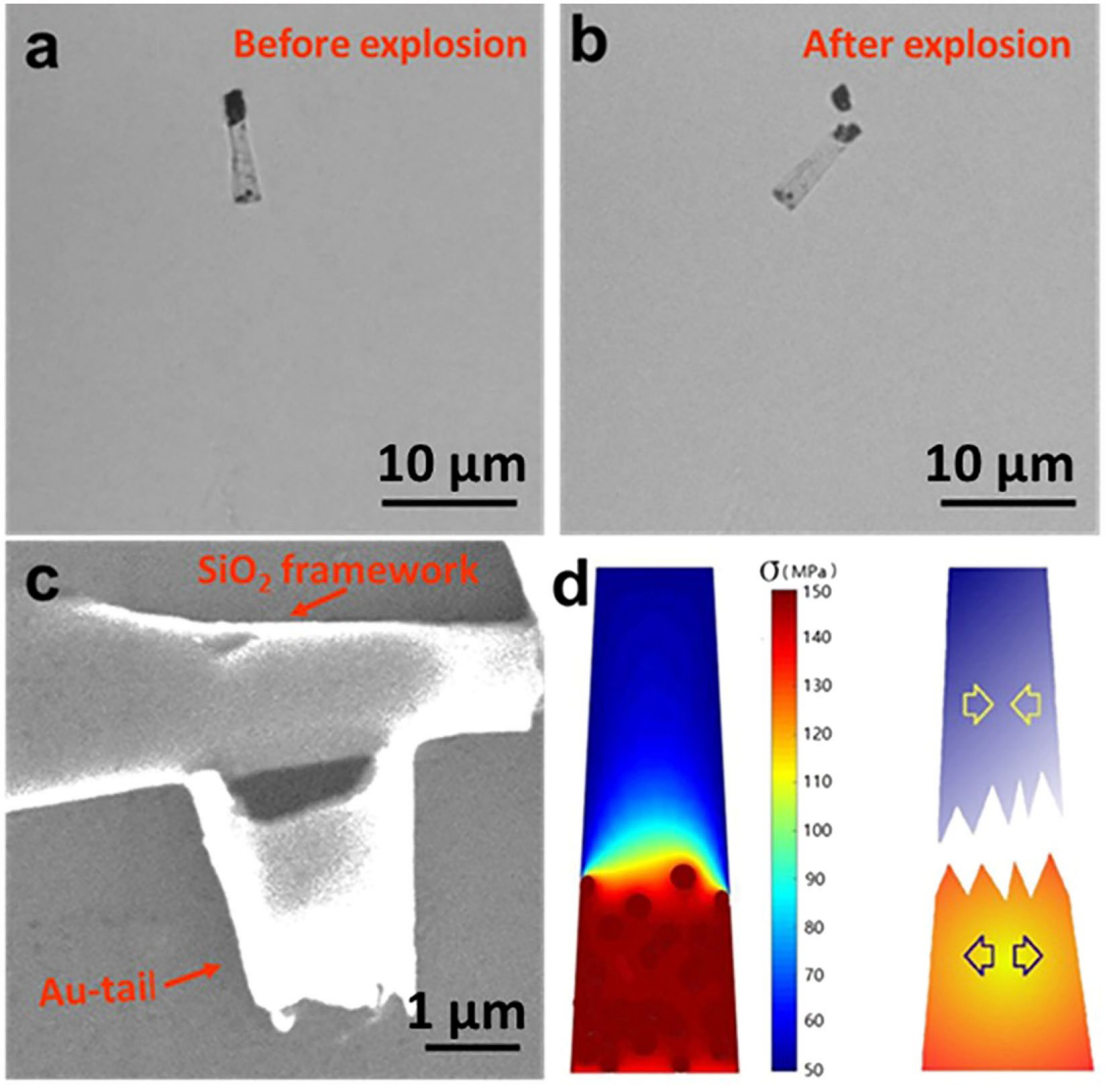
Time-Lapsed Images from Supplymentary Video 5 of A Torpedo Motor. (**a**) Au-tailed torpedobefore explosion. (**b**) Au-tailed torpedobefore explosion. (**c**) The SEM image of a torpedo after explosion. (**d**) Theoretical simulation of the NIR-triggered explosion of torpedo. The thermal stress distribution for the Au-tailed torpedo (left); Schematic effect of thermal stress: The tubular part with high temperature (the orange part) expands, the cold tubular part contracts (the blue part).

Besides the NIR-triggered movement, the torpedoes also allow for the on demand explosion upon high power of NIR irradiation. Once the output power of NIR laser was beyond 32.3 J cm^−2^, one can see that an intact torpedo (Fig. 6a captured from Suppplementary Video 5) instantaneously exploded into two big pieces (Fig. 6b captured from Supplementary Video 5). The SEM image of the corresponding broken torpedo displays that the breakage was mainly occurred in the junction of AuNPs clusters-functionalized tail and silica framework (Fig. 6c). The torpedo possesses a complex structure of three layers of tubular silica and an inner layer of loosely combined AuNPs clusters which mainly covers the wide opening end. Upon the irradiation of NIR laser, the temperature of the AuNPs clusters rapidly arises owing to photo-thermal effect, while the temperature of the uncovered remains a relative low temperature. In this case, a heated area expands its volume since the water brings the heat away through the convective flow (Fig. 6d, right scheme). Given that the torpedo motors have the same physical property as the conventional glass tube within tolerable fluctuations. To give a rough estimation on the explosion of torpedo, the Young’s modulus of the torpedoes was taken as the same as glass, E = 7.3*10^10^ Pa, and the linear thermal expansion coefficient is β = 7*10^−6^/°C. Therefore, the thermal stress that breaks the torpedoes can be roughly computed by a linear approximation equation^49, 50^,

4 $$\sigma ={\rm{E}}\beta {\rm{\Delta }}T$$

Here ΔT is the temperature difference between the hot centre and the cold edge. Figure 6d shows the distribution of thermal stress along the torpedo body. The temperature difference induces the thermal stress on the interface between the cold area and hot area. Once this thermal stress exceeds the critical maximal strength of the torpedo, it would break into pieces. In other words, the explosion of torpedo motor is caused by the highly inhomogeneous thermal stress distribution across the torpedo body. Note that a low thermal stress only causes single crack in the beginning and leads to a few broken pieces, while a high thermal stress would result in more pieces.

**Figure 7:**
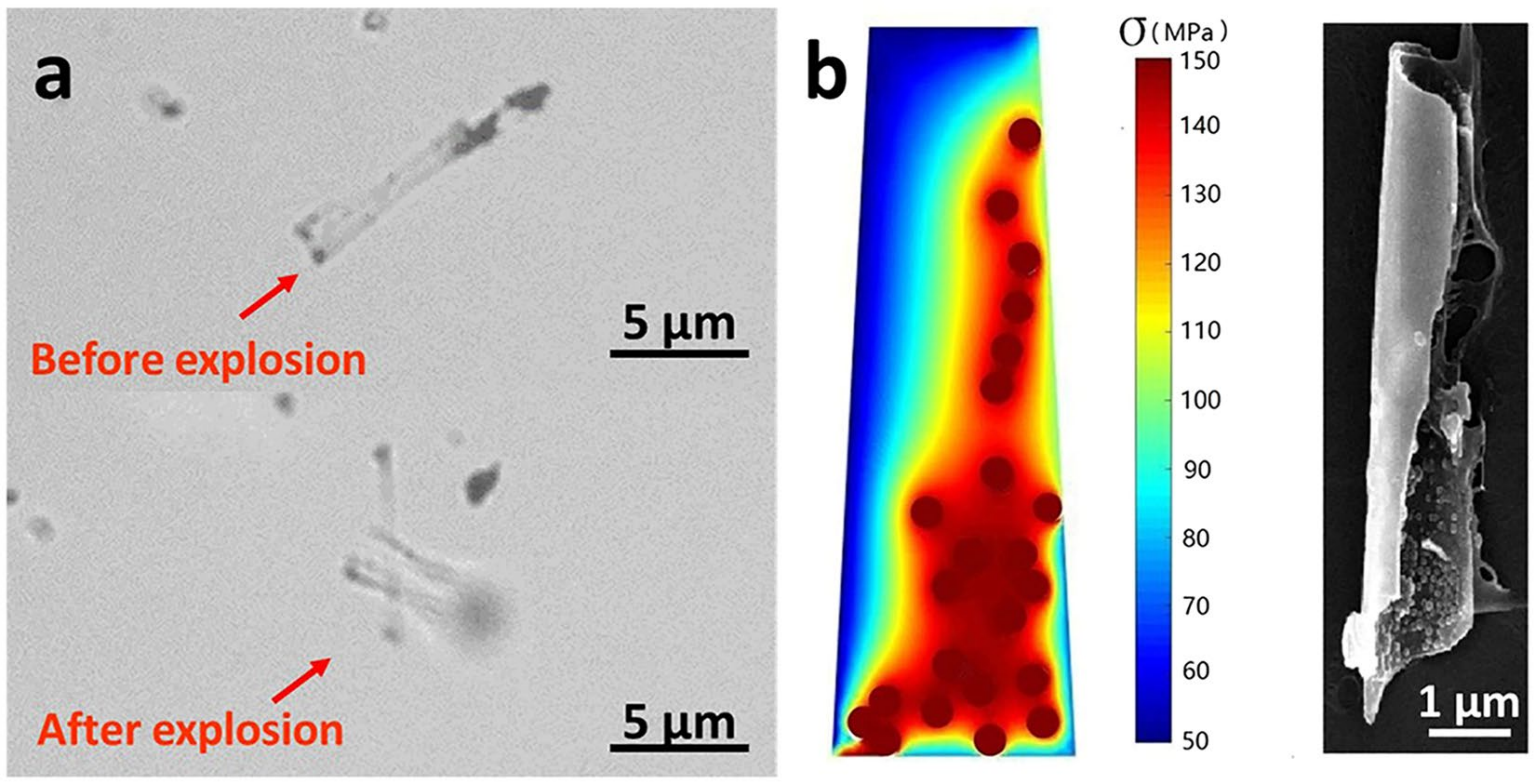
Gold Nanoparticles Longitudinal-Functionalized Silica Torpedo Exploded under NIR Laser Irradiation. (**a**) The time-lapsed image captured from Supplementary Video 6. (**b**) The thermal simulation and SEM image after explosion.

Finally, the AuNPs distribution inside the torpedoes could be roughly controlled by modulating the repeated wet-dry process. Figure 7a, captured from Supplementary Video 6, shows a longitudinally AuNPs clusters-functionalized torpedo, and under exposure of NIR laser, the torpedo exploded into scattered strip along the boundary of the AuNPs clusters. There exists an obviously inhomogeneous thermal stress along the boundary. Figure 7b further shows the computed thermal stress distribution on the torpedo by using the above thermal stress equation. The thermal stress suddenly jumps along the boundary line between the Au coated part and uncovered part. The SEM image of the exploded torpedo fragments reveals the highly inhomogeneous distribution of AuNPs clusters on the inner wall of torpedo (Fig. 7b). It means that the explosive position of the torpedoes could be tuned by assembling AuNPs in a controlled manner.

In summary, we produced a fuel-free torpedo motor which performs stable movement in a straight line in various media, such as pure water, salt solution, PBS, and cell cultured media. The velocity of torpedo motors can be controlled by operating the power of the NIR laser, and the driving mechanism of the NIR-activated torpedo motors is self-thermophoresis. The asymmetric geometry ensured its stable trajectory in a straight line, which is also confirmed by fluid dynamic simulation. Compared with previously NIR-powered synthetic motors propelled by the focused laser, the equally photons-distributed NIR laser in TP-CLSM provide a more controllable and stable velocity. The explosion of torpedoes can be controlled by different deposited spot. Such free-fuel torpedoes provide a path to develop a biocompatible synthetic motor for biomedical application.

## Methods

### Materials

Commercial porous polycarbonate membranes with the cone-shaped pore wide diameter of 3 μm were purchased from Whatman Corp. The SiCl_4_ was purchased from Acros. CCl_4_, CH_2_Cl_2_, HAuCl_4_·4H_2_O, citric acid monohydrate, methanol and ethanol were used without further purification. The water used in all experiments was prepared in a Milli-Q purification system with the resistivity higher than 18.2 MΩ cm^−1^.

### Preparation of gold nanoparticles (AuNPs)

Citrate-stabilized gold nanoparticles were synthesized according to previous reports^32^. 50 mL of a citric acid (2.2 mM) solution was placed in a three-neck round bottom flask and heated to 100 °C. Subsequently, 1 mL of 25 mM HAuCl_4_ solution was added and the reaction mixture was heated to 100 °C for 3.5 min before it was allowed to cool to room temperature.

### Preparation of gold film-functionalized torpedoes

The silica tubes were synthesized according to the previous reports^42^. Commercially available porous polycarbonate membranes with the cone-shaped pore wide diameter of 3 μm were used. A membrane was immersed in a SiCl_4_ solution for 3 min and quickly washed with CCl_4_ to remove the reagent from the faces. The membrane was then placed in a beaker with a fresh portion of CCl_4_ for 20 min to remove the free SiCl_4_ from the pores. Finally, the membrane was soaked in a CCl_4_/MeOH 1:1 (2 min) and EtOH (5 min) to displace CCl_4_, and dried in a vacuum jar with a residual pressure of 10^−7^ mbar at 100 °C. Then the membrane was immersed in deionized water for 6 min, washed in a beaker with MeOH (2 min), and dried in a vacuum jar (10^−7^ mbar) at 100 °C for 30 min. After each adsorption and hydrolysis step, a SiO_2_ layer was developed. When 3 SiO_2_ layers were obtained, several drops (rougly 50 μl) of Au nanoparticles drip on the wide-opening surface of membranes. Then the Au nanoparticle solution-caped membrane dried in a vacuum at 100 °C for 30 min, repeating the procedure for 3 times. The top and bottom surface of membrane template were polished using a wet cotton swab with alumina powder (average mech size of 30 μm) to obtain the single tubular microtorpedoes. Subsequently, the template were dissolved in CH_2_Cl_2_, the resulting solution was washed with CH_2_Cl_2_ for 5 times. The resulting torpedoes were collected by centrifugation at 3000 rpm for 5 min. followed by dispersing in ethanol and water. The torpedoes solution was then stored at 4 °C for further experiment.

### Motion experiments

A drop of a gold film-functionalized silica torpedoes solution was placed on a glass slide. A cover slide was then put on it and sealed with nail oil. Then two-photon confocal laser scanning microscopy (TP-CLSM, Olympus FV1000) was employed to record the movement of gold film-functionalized silica torpedoes. A self-coded software based on MAT-LAB 2012 was utilized to analysis and track the motorial behaviour of Au-caped silica torpedoes.

### Characterization

SEM and EDX images were collected by a Quanta 200FEG instrument at an operating voltage of 20 k eV. A drop of Au-caped silica torpedoes solution was dripped on a silicon wafer for the test. TEM analysis was performed on a JEOL 2100 microscope operated at 100 kV. The samples were prepared on a carbon-coated copper grid.

## Electronic supplementary material

Supplementary Information

Supplementary video 1

Supplementary video 2

Supplementary video 3

Supplementary video 4

Supplementary video 5

Supplementary video 6
