# Supplementary material for: A Light-Activated Explosive Micropropeller
Source: Sci Rep. 2017 Jul 4;7:4621. doi: 10.1038/s41598-017-04908-x (PMC5496919; doi:10.1038/s41598-017-04908-x)
Supplement: Supplementary file 1 — Supplementary Information [file 41598_2017_4908_MOESM1_ESM.docx]

**Supporting Information**

**A Light-Activated Explosive Micropropeller**

Qianlan Rao,^1^ Tieyan Si,^1^ Zhiguang Wu,^1,2,*^ Mingjun Xuan,^1^ Qiang He^1,*^

^1^ Key Laboratory of Microsystems and Microstructures Manufacturing, Micro/Nanotechnology Research Center, Harbin Institute of Technology, Yikuangjie 2, Harbin 150080 (China)

^2^ Max Planck Institute for Intelligent Systems, Heisenbergstrasse 3, Stuttgart 70569 (Germany)

E-mail: zhiguangwu@hit.edu.cn; qianghe@hit.edu.cn

**TABLE OF CONTENTS**

Supplementary Figure S1

Supplementary Video 1

Supplementary Video 2

Supplementary Video 3

Supplementary Video 4

Supplementary Video 5

Supplementary Video 6

**
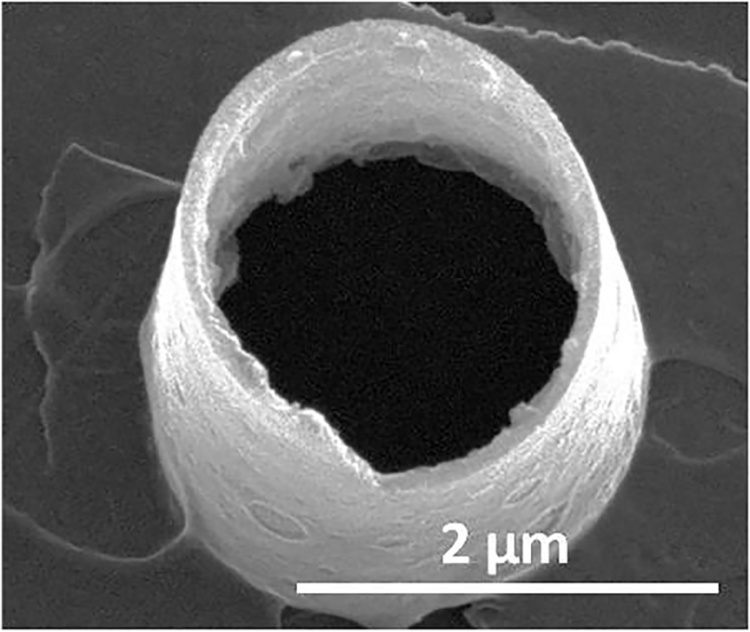
**

**Supplementary Figure S1** The top view SEM image of the bare torpedo.

**Supplementary Video 1** Nearly straight linear movement of the torpedo in pure water under NIR laser power of 5.5 J cm^-2^.

**Supplementary Video 2** Autonomous motion of the torpedo in PBS under NIR laser power of 5.5 J cm^-2^.

**Supplementary Video 3** Autonomous motion of the torpedo in seawater under NIR laser power of 5.5 J cm^-2^.

**Supplementary Video 4** Autonomous motion of the torpedo in cell culture media under NIR laser power of 5.5 J cm^-2^.

**Supplementary Video 5** Explosion of torpedo into two parts in water under NIR laser power of 32.3 J cm^-2^.

**Supplementary Video 6** Explosion of torpedo along the longitudinal axis in water under NIR laser power of 32.3 J cm^-2^.
